# Rapid, repeated, low-dose challenges with SIVmac239 infect animals in a condensed challenge window

**DOI:** 10.1186/s12977-014-0066-z

**Published:** 2014-08-14

**Authors:** Justin M Greene, Andrea M Weiler, Matthew R Reynolds, Brian T Cain, Ngoc H Pham, Adam J Ericsen, Eric J Peterson, Kristin Crosno, Kevin Brunner, Thomas C Friedrich, David H O’Connor

**Affiliations:** Department of Pathology and Laboratory Medicine, University of Wisconsin-Madison, Madison, Wisconsin 53706 USA; Wisconsin National Primate Research Center, University of Wisconsin-Madison, Madison, Wisconsin 53715 USA; Department of Pathobiological Sciences, University of Wisconsin, Madison, WI USA

## Abstract

**Background:**

Simian immunodeficiency virus (SIV) infection of nonhuman primates is the predominant model for preclinical evaluation of human immunodeficiency virus (HIV) vaccines. These studies frequently utilize high-doses of SIV that ensure infection after a single challenge but do not recapitulate critical facets of sexual HIV transmission. Investigators are increasingly using low-dose challenges in which animals are challenged once every week or every two weeks in order to better replicate sexual HIV transmission. Using this protocol, some animals require over ten challenges before SIV infection is detectable, potentially inducing localized immunity. Moreover, the lack of certainty over which challenge will lead to productive infection prevents tissue sampling immediately surrounding the time of infection.

**Findings:**

Here we challenged Mauritian cynomolgus macaques with 100 50% tissue culture infectious doses (TCID_50_) of SIVmac239 intrarectally three times a day for three consecutive days. Ten of twelve animals had positive plasma viral loads after this challenge regimen.

**Conclusions:**

This approach represents a straightforward advance in SIV challenge protocols that may avoid induction of local immunity, avoid inconsistent timing between last immunization and infection, and allow sampling immediately after infection using low-dose challenge protocols.

**Electronic supplementary material:**

The online version of this article (doi:10.1186/s12977-014-0066-z) contains supplementary material, which is available to authorized users.

## Findings

Developing both cures and vaccines for HIV requires a clear understanding of early events after infection when HIV seeds the latent reservoir and decimates the mucosal immune system; however, it is nearly impossible to determine, with certainty, the sequence of the infecting virus or exactly when an individual was infected. These complications mean it is difficult to study early events after infection or understand how the virus evolves in response to immune pressure during early infection. Simian immunodeficiency virus (SIV) infection of nonhuman primates (NHPs) is a well-established model of HIV [1]. NHPs enable preclinical studies of the safety and efficacy of HIV vaccines or therapeutic HIV interventions. Thus, SIV studies in NHPs allow for prospective experiments with well-characterized virus stocks, invasive sampling, and known times of infection.

Until recently, investigators used extremely high-dose challenges that did not recapitulate certain facets of sexual HIV transmission. HIV infection is typically initiated by a single virus that replicates in the tissues and then spreads systemically [2,3]. Moreover, productive infection is a rare event; it is estimated that people are infected in only approximately 1 of every 500 heterosexual contacts [4]. When macaques are challenged with large amounts of virus at mucosal tissues, multiple viruses initiate infection [3]. Such high-dose challenges could mask the protective effects of an otherwise efficacious vaccine [5]. More recently, investigators have developed a low-dose challenge model in which macaques are repeatedly challenged with a low-dose of SIV [3,6]. The protocols are designed so that multiple challenges (typically three to four, given 1 week apart) are required to infect most unvaccinated macaques. Consequently, these challenges can take multiple weeks before all animals are productively infected. While SIV transmission from low-dose challenge protocols more closely models heterosexual HIV transmission, these approaches have several drawbacks. It is impossible to know in advance which challenge will initiate productive infection, precluding studies that require exact timing of infection or tissue sampling in the hours or days after infection. Additionally, repeated exposures to HIV can lead to the development of HIV-specific local and systemic immunity [7]. It is therefore likely that similar immune effects could occur over time in macaques exposed to SIV but not productively infected. In macaque vaccine trials using typical low-dose challenge protocols, it may be difficult to tease apart vaccine-induced immune responses from immune responses resulting from repeated exposures to SIV. Certain studies may benefit from avoiding this potential induction of immunity while other studies might seek to replicate it.

Our lab also performs adoptive transfers between macaques to examine protective immune responses; however, the limited persistence of donor cells provides a narrow window for SIV challenges in order to assess the protective capacity of the transferred cells [8–10]. We were concerned a high-dose challenge might mask antiviral effects exerted by the adoptively transferred cells. The clearance kinetics of our donor cells led us to investigate whether a low-dose challenge was feasible in a shorter timeframe. Passive antibody transfer studies might suffer from similar issues. For example, passively transferred neutralizing antibodies may have a short half-life *in vivo* and thus a short window of potential activity [11,12]. Therefore, we developed a protocol to challenge animals intrarectally with a low-dose of SIV multiple times in a short duration, an approach termed “rapid, repeated, low-dose” infection (RRLD). All animals included in these studies were Mauritian origin cynomolgus macaques and these studies were approved by the Institutional Animal Care and Use Committee.

**Figure 1:**
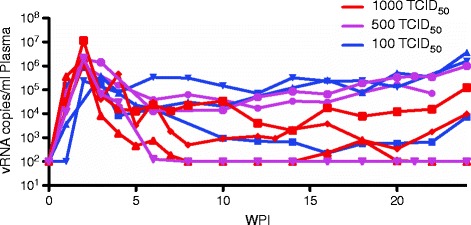
**Plasma vRNA per ml of plasma in animals challenged with indicated dose of SIV.**

First, we titrated a stock of SIV in nine animals. These animals were challenged in groups of three using 1000 TCID_50_, 500 TCID_50_, or 100 TCID_50_ (Figure 1). Animals were challenged every two weeks during this titration. The animals challenged with 1000 TCID_50_ were all infected after a single challenge. The 500 TCID_50_ dose infected two animals after a single challenge and one animal after two challenges. The 100 TCID_50_ challenge infected one animal with a single challenge, one animal after two challenges, and one animal after four challenges. All animals were monitored for 24 weeks. Two animals that share the M7 MHC haplotype maintained control of viral replication to undetectable levels. Given the results of the titration, we used 100 TCID_50_ for our low-dose challenges.

**Figure 2:**
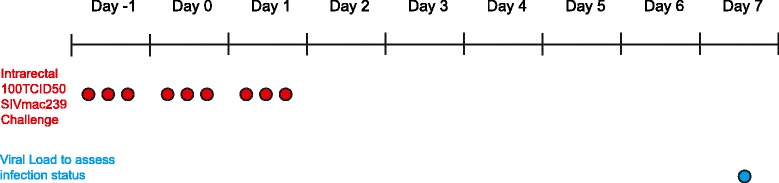
**Rapid repeated low-dose challenge protocol.** Animals were challenged three times a day for three consecutive days.

The RRLD challenges were piloted in two MHC-defined Mauritian cynomolgus macaques. Both animals were challenged with 100 TCID_50_ SIVmac239 intrarectally at four-hour intervals, three times a day for three consecutive days (Figure 2). Specifically, we delivered virus to the WNPRC at 0700, 1100, and 1500. Virus was introduced by a 1 ml syringe to the rectum and animals remained elevated to allow for virus interaction with host cells. Challenges were typically performed on Tuesday, Wednesday, and Thursday. These animals were lightly anesthetized at each challenge. The animals were anesthetized for at least 10 minutes after drug delivery and allowed to recover quickly after the procedure was finished to provide for adequate food intake during the challenge interval. Animals were anesthesized with a ketamine/dexmedetomidine mixture with an atipamezole reversal. We used 3 mg/kg ketamine and 0.015 mg/kg dexmedetomidine given intramuscularly (IM). This differed from the standard dose of 5 mg/kg ketamine and 0.015 mg/kg dexmedetomidine. At the end of the procedure 0.15 mg/kg atipamezole was given IM as a reversal for the dexmedetomidine. Supplemental food was also provided between multiple anesthetic events, and food intake/weight were monitored closely by WNPRC veterinary staff. Most animals responded well to the lower dose of ketamine. They remained anesthetized for the entire procedure and recovered quickly following the atipamezole reversal. Animals that were not sufficiently anesthetized at the starting dose were given a higher dose of ketamine starting at 4 mg/kg to the standard dose of 5 mg/kg.

**Figure 3:**
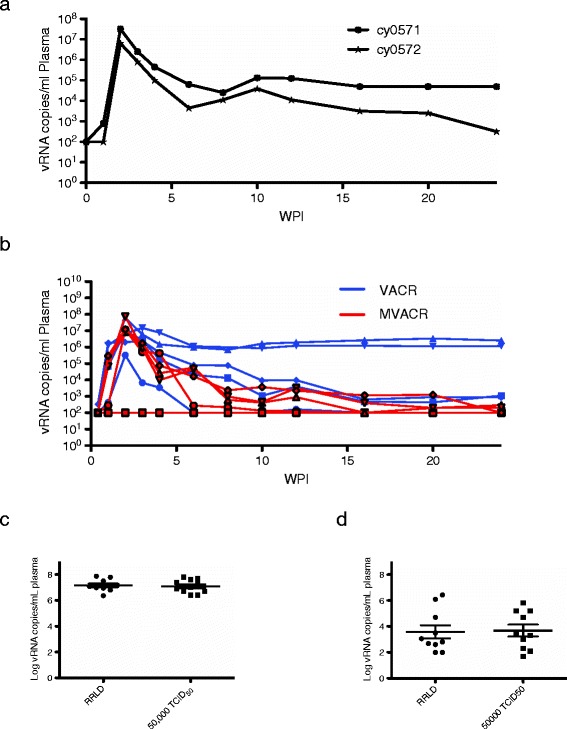
**Viral loads were measured in SIV-challenged animals.** Plasma vRNA per ml of plasma. **a)** in two pilot animals challenged by RRLD and **b)** in ten animals that received cells from donor macaques and were then challenged using the RRLD protocol. These ten animals include both vaccinated animal cell recipients (VACR) and mock-vaccinated animal cell recipients (MVACR). **c)** Peak viral loads for animals challenged by RRLD protocol or standard single 50,000 TCID50 challenge. **d)** Viral loads from RRLD and standard single 50,000 TCID50 challenge at 16-18 weeks postinfection. The high-dose challenge viral loads have been published in a previous manuscript [10].

We monitored the animals for 24 weeks after infection. We arbitrarily established the second day of challenges as day 0. Animals developed normal acute phase viral loads that reached 3.3 × 10^7^ and 6.9 × 10^6^ copies vRNA/ml plasma (Figure 3a). These viral loads are comparable to Mauritian cynomolgus macaques challenged with SIV with a single high-dose of SIV (Figure 1 and Data not shown).

Next we challenged 10 animals as part of an adoptive transfer study (Additional file 1: Figure S1). These animals were recipients of cells from either SIVmac239Δnef vaccinated or mock-vaccinated donors. A day after transfer, we began challenging animals with SIVmac239 using the RRLD challenge protocol. The aim of this study was to determine whether cells transferred from vaccinated macaques could prevent infection or reduce viral loads after challenge. Eight of the ten animals were infected after one round (nine total challenges in three days) of RRLD challenges (Figure 3b). One animal, cy0418, was infected with SIVmac239Δnef as a result of the transfer but did not get infected by SIVmac239, as determined by discriminating qPCR (Additional file 2: Figure S2). These animals developed comparable viral loads to historic controls challenged with higher doses of SIV (Figure 3c,d). In addition, we did not observe lower viral loads in animals that received cells from vaccinated donors (Figure 2 and Data not shown). At 24 weeks post-infection, animals that received cells from mock-vaccinated donors exhibited reduced viral loads compared to the animals that received cells from vaccinated macaques. This may be an effect of the adoptive transfer; however, all of these animals were enriched for the M1 MHC haplotype that is correlated with SIV control [13]. Thus, these viral load differences may reflect coincidental enrichment for controllers in the animals that received cells from mock-vaccinated donor animals. It is possible that adoptive transfer may have led to immune activation and increased target cell susceptibility to infection; however, we do not have direct evidence that this occurred and both pilot animals were infected after a single round of challenges during the pilot study.

Previous studies of low-dose challenges have demonstrated a bottleneck in the number of transmitted variants compared to high-dose challenges [14]. We challenged with a clonal SIVmac239 stock; however, even a clonal stock may contain very low frequency variants [15]. Therefore, we expected that low-frequency variants might still be detectable in animals post-challenge and that these variants might help determine the number of transmitted viruses. We acquired full-length SIV sequences from four of the 12 macaques at one and two weeks post-infection by MiSeq. We examined 5 sets of 50,000 reads subsampled from each macaque’s total reads and looked for synonymous or non-synonymous SNPs present in greater than 1% of sequence reads in at least three of the subsampled alignments. All animals had at least one SNP at one week post-infection and cy0571 had 19 SNPs (data not shown). The large number of SNPs detected in cy0571 may reflect re-sequencing artifacts because cy0571 had plasma viral loads below 1000 copies vRNA/ml plasma. The majority of the variants in cy0571 were undetectable by 2 weeks post-infection while other animals exhibited similar numbers of SNPs at one and two weeks post-infection. The contraction in viral genetic diversity in cy0571 suggests that sequencing virus at time-points after one-week post-infection may underestimate diversity of the viruses that initiate and seed infection. We compared the number of SNPs detected in the RRLD animals to two animals challenged with 7,000 TCID_50_. We did not detect differences between the number of SNPs in animals challenged with a high-dose or low-dose of SIV at one week post-infection; however, there were limited number of animals in this control group.

These results demonstrate that it is possible to condense low-dose challenge regimens into a three-day window. In the current approach, the specific challenge(s) that initiate infection are not known. Individuals who are interested in determining which challenge resulted in productive infection can utilize distinctly barcoded viruses [16]. Using a uniquely barcoded virus in each challenge should allow for a clear delineation of which challenge or challenges infected the animals. We expect that these results could be extrapolated to other models of HIV infection including the more pathogenic infection of rhesus macaques; however, labs would likely need to titrate their stocks prior to beginning their experiments. Ultimately, these studies demonstrate that it should be possible to proceed more rapidly through low-dose challenge regimens.

We foresee several circumstances in which this novel challenge protocol may be valuable. First, investigators are performing *in situ* studies of animal tissue shortly after challenge when SIV is replicating in the tissues; however, these studies require that animals be euthanized after challenge and necessitate high-dose challenges to ensure researchers can capture viral replication shortly after infection [17–19]. By rapidly performing multiple challenges, investigators can be relatively confident that an animal was productively infected; thus, they can perform invasive sampling or euthanize an animal and study early acute events while using a low-dose challenge.

Second, investigators performing microbicide and vaccine trials are frequently interested in studying the longitudinal efficacy of the relevant intervention. Currently, low-dose challenge protocols yield indeterminate intervals between microbicide application or vaccination and infection because animals may need to be challenged on multiple occasions spanning several weeks or months. The RRLD protocol provides a much more clearly defined duration between intervention and challenge.

Finally, the RRLD protocol could be adapted to use ultra-low-dose challenges. As discussed above, HIV transmission is rare, with only 1 in 500 heterosexual contacts leading to HIV infection [4]. Low-dose challenges more accurately duplicate the biology of HIV transmission because, typically, only one virus leads to systemic infection, but current low-dose challenges still result in infection at a far higher rate than is actually observed in humans. It is conceivable that these low-dose challenges are too stringent, and may mask the protective efficacy of vaccines in pre-clinical trials. It should be possible to further titrate the virus challenge stock until only 1 in every 100 challenges results in a productive infection. Animals could be challenged with an ultra-low-dose of SIV by RRLD protocol for 9-10 weeks until all animals are infected. For example, animals could be challenged, 9 times over three days and similarly re-challenged every 2-3 weeks. Using this rapid, repeated, ultra-low-dose protocol, the number of challenges to infect animals is an additional, measureable parameter of vaccine efficacy. This regimen would reintroduce elements of traditional low dose challenges like potential immune induction from repeated SIV exposures, but might provide a valuable model of HIV infection. It would also require a large number of anesthetic events, but animals are lightly anesthetized for these procedures and the challenges could be performed over a longer period of time. Ultimately, the RRLD regimen may more accurately recapitulate sexual HIV transmission than a single high-dose challenge while allowing for more intensive sampling during the very early stages of viral replication and known durations between intervention and infection. Certain studies may benefit from this novel challenge protocol.

## Additional files

Additional file 1: Figure S1. Animals included in the adoptive transfer experiment. Additional file 2: Figure S2. Results of the discriminatory qPCR designed to detect wild-type SIV or nef deleted SIV.
